# Anti-ganglioside Complex IgM Antibodies in Multifocal Motor Neuropathy Post-influenza Vaccination

**DOI:** 10.7759/cureus.22918

**Published:** 2022-03-07

**Authors:** Krithika Suresh, Preethi Mereddy, Nicholas Lanciano, Md Didar Ul Alam

**Affiliations:** 1 Internal Medicine, Conemaugh Memorial Medical Center, Johnstown, USA; 2 Neurology, Conemaugh Memorial Medical Center, Johnstown, USA

**Keywords:** paresthesia, intravenous immunoglobulin (ivig), influenza vaccine, neuropathy, multifocal motor neuropathy

## Abstract

Multifocal motor neuropathy (MMN) is a peripheral nerve disorder characterized by progressive, predominantly distal, asymmetric limb weakness with minimal or no sensory impairment, and characterized by the presence of antibodies (30-80% cases), mostly IgM, to the gangliosides, mainly ganglioside monosialic acid (GM1). We describe a case of MMN in a patient who developed symptoms of paresthesia and extremity weakness a few days after receiving the influenza vaccine and was found to have high titers of anti-GM1 IgM antibody levels. He was initially treated with intravenous immune globulin (IVIG) which is one of the mainstays of treatment but relapsed and was then successfully treated with plasma exchange.

## Introduction

Multifocal motor neuropathy (MMN) is a recently identified rare peripheral nerve disorder characterized by progressive, predominantly distal, asymmetric limb weakness mainly affecting upper limbs, with minimal or no sensory impairment, and by the presence on nerve conduction velocities of multifocal, persistent partial conduction blocks on the motor but not sensory nerves. Antibodies, mostly IgM, to ganglioside monosialic acid (GM1), and, less frequently, GM2 and GD1a, are detected in the patients’ serum. This helps to aid in the diagnosis of this disease [1].

## Case presentation

A 66-year-old male with a past medical history of tobacco use presented with right greater than left upper extremity paresthesia as well as bilateral lower extremity weakness (right greater than left), numbness, and tingling in his fingertips. He also reported a sensation of blurred vision in the left eye towards the evening with some drooping. He had reported receiving a flu shot about 10 days prior to presentation. He denied any urinary symptoms, dysphagia, shortness of breath, fever, chills, chest pain, or facial droop. Initial vitals recorded on presentation were stable.

Physical exam was remarkable for decreased overall muscle bulk, motor exam showed intact strength 5/5 bilateral upper and lower extremities. Upper extremity reflex testing including biceps, triceps, and brachioradialis were 1/4 bilaterally; patellar and Achilles’ tendon reflexes were absent bilaterally with fasciculations noted more prominent over the right lower extremity. Sensory exam was remarkable for diminished vibration sense over toes bilaterally and was intact at the knees and fingertips bilaterally. Light touch was preserved throughout the lower extremities.

Lab workup including complete blood count (CBC), comprehensive metabolic panel (CMP), Lyme titers, creatine kinase (CK), aldolase, vitamin D, and B12 levels were within normal range. Myasthenia gravis antibody panel was negative. MRI head without contrast showed no acute intracranial abnormality. As the patient was afebrile and CBC was normal and no evidence of mental status changes cerebrospinal fluid (CSF) analysis was not done. He was further evaluated with an electromyography (EMG) with motor and sensory nerve studies as shown in Table 1 and Table 2. 

**Table 1:** Electromyography (EMG) showing motor nerve study C.V.- Conduction velocity; Amp-amplitude; mV- millivolt; ms-millisecond

Nerve and Stimulation site	Latency (ms) Ref: 2.3-7.2	Duration (ms)	Amp (mV) Ref:3-20	C.V. (m/s) Ref: 50-70
Right median nerve				
Wrist	4.3	6.1	6.9	
Elbow	8.2	6.8	5.6	60.0
Right ulnar neve				
Wrist	2.8	33.8	0.433	
Elbow	5.5	7.0	5.2	48.5
Right peroneal nerve			Ref: (5-20)	Ref: (40-70)
Ankle	3.7	5.6	6.3	
Fib head	11.0	6.6	5.1	46.4
Pop fossa	13.7	7.1	5.2	37.5
Right Tibial nerve				
Ankle	3.8	3.4	3.8	
Pop fossa	12.1	4.5	2.8	52.8

**Table 2:** Electromyography (EMG) showing sensory nerve study MCV- Microvolt

Nerve and Stimulation site	Latency (ms) Ref: 2.3-4.4	Peak latency (ms) Ref: 2.3-3.5	Amp (MCV) Ref: (6-90)
Right median nerve			
Thumb	2.6	3.5	25.3 (20-90)
Right Ulnar Nerve			
5^th^ digit	2.3	3.2	30.0 (17-90)
Right sural nerve			
Mid-calf	2.8	3.7	15.3 (6-90)

EMG was interpreted as abnormal due to prolonged latency of right median compound muscle action potential (CMAP) and right peroneal CMAP, low conduction velocity (C.V.) of right ulnar CMAP, and low C.V. right peroneal CMAP. Sensory nerve action potential (SNAP) was largely normal. It was concluded that there was electrophysiologic evidence of a demyelinating motor neuropathy with evidence of conduction block, raising the possibility of atypical Guillain-Barre syndrome (GBS) or other acute inflammatory demyelinating polyneuropathy (AIDP) without evidence of sensory involvement. However, due to his motor involvement as evidenced by EMG studies that were more prominent in the upper than lower extremities, there was also a concern for multifocal motor neuropathy (MMN).

He was tested for the anti-GM1 antibody and his anti-GM1 IgM was positive with >100% (reference range 0-30) and negative for the anti-GM1 IgG antibody. He was treated with intravenous immune globulin (IVIG) at 0.4 g/kg daily for five days. He completed the course of IVIG with marked improvement of symptoms. He was discharged to acute inpatient rehabilitation with Physical Therapy (PT) and Occupational Therapy (OT) and was also recommended to follow up as an outpatient. He was scheduled to receive 0.5 g/kg IVIG every 28 days following discharge. About a month later, prior to his scheduled IVIG infusion, the patient’s clinical condition deteriorated at acute inpatient rehabilitation necessitating him to be readmitted to the hospital. During that hospitalization, he was treated with plasmapheresis (five rounds of 3000 mL exchanges with 5% albumin) and subsequently discharged to acute inpatient rehabilitation.

It was noted then that he was unable to ambulate independently following the second hospitalization. However, he was continued on monthly plasmapheresis (one round of 3000 mL exchanges with 5% albumin every 28 days) and participated in aggressive acute inpatient physical and occupational therapy rehabilitation. Two months later, he was able to ambulate without assisted devices. Three months later, he was able to ambulate normally with only sensations of pins and needles in his hands, but with a normal neurologic examination. He has been continued on monthly plasmapheresis (one round of 3000 mL exchanges with 5% albumin every 28 days) and his disease has remained clinically quiescent for over a year, from the time of initial presentation.

## Discussion

Multifocal motor neuropathy (MMN) is a rare inflammatory neuropathy characterized by slowly progressive, asymmetric distal limb weakness without sensory loss [2]. The diagnosis can appear clinically and electrophysiologically like Lewis-Sumner syndrome (LSS), but with LSS typically not having positive anti-GM1 antibodies and being more responsive to treatment [3].

Post-influenza vaccine Guillain-Barre syndrome (GBS) is a much more frequently reported entity as compared to MMN post-influenza vaccination. Meta-analysis and systematic reviews did reveal that GBS is a rare adverse effect of influenza vaccination; however, higher rates of GBS were seen with the actual influenza virus. The typical presentation of GBS post-influenza vaccine was about seven to 14 days from vaccination [4]. There are also case reports of LSS in the setting of influenza [5]. To our knowledge, there are no reports of MMN occurring in relation to the influenza vaccine or the influenza virus itself.

There have been associations with anti-GM1 antibodies playing a role in the pathogenesis of MMN suggesting an autoimmune basis of the disease process as well as a robust response to immunomodulatory treatment. As this condition can mimic other neurological disorders including GBS-motor variant, Lewis-Sumner syndrome, and other motor neuron diseases it is important to recognize this entity as management would potentially differ [6].

MMN is a rare and disabling disease and several studies suggest strong immune-mediated pathogenesis mainly because of the presence of IgM anti-GM1 serum antibodies and the positive response noted to treatment with IVIG. IVIG remains the treatment of choice for MMN and is recommended as first-line therapy based on multiple studies and randomized trials [7]. IVIG is commonly used in the treatment of immune-mediated disorders. While patients do remain stable with maintenance IVIG treatment, most patients will slowly deteriorate over many years [8].

There are some case reports and non-randomized trials suggesting variable results from therapeutic or adjunctive use of other immunosuppressive or immunomodulatory agents such as cyclophosphamide, cyclosporine, methotrexate, azathioprine, interferon beta-1a, and rituximab. Of these, cyclophosphamide and rituximab are the immune treatments that have shown some benefits in case reports [9].

Our case was rare, as we found that the patient developed MMN with high anti-GM1 IgM antibody levels a few days after influenza vaccination. Our patient also had motor impairment as evidenced by EMG studies. He was treated with IVIG with an improvement of his symptoms initially; however, later deteriorated and required plasmapheresis which was continued for maintenance therapy. Although the literature review showed IVIG as a mainstay of treatment for MMN, our patient seemed to have a positive response while on plasmapheresis, after failing IVIG and has put his disease currently into remission.

## Conclusions

Post-influenza vaccination MMN is a rare entity, which should be a differential in patients presenting with rapidly progressive neurological symptoms and features of GBS as rapid diagnosis and appropriate treatment can have better clinical outcomes. A thorough neurological examination and testing with EMG could aid in prompt diagnosis and management with anti-GM1 antibodies where appropriate. Our patient had reported improvement of symptoms after he received treatment with IVIG, but later relapsed requiring treatment with plasmapheresis, and has remained currently in remission.
